# The energy and mass balance of a continental glacier: Dongkemadi Glacier in central Tibetan Plateau

**DOI:** 10.1038/s41598-018-31228-5

**Published:** 2018-08-24

**Authors:** Liqiao Liang, Lan Cuo, Qiang Liu

**Affiliations:** 1 Key Laboratory of Tibetan Environment Changes and Land Surface Processes, Institute of Tibetan Plateau Research, Chinese Academy of Sciences, Beijing, China; 2 Center for Excellence in Tibetan Plateau Earth Sciences, Chinese Academy of Sciences, Beijing, China; 3 University of Chinese Academy of Sciences, Beijing, China; 4 State Key Laboratory of Water Environment Simulation, School of Environment, Beijing Normal University, Beijing, 100875 China; 5 Key Laboratory for Water and Sediment Sciences, Ministry of Education, School of Environment, Beijing Normal University, Beijing, 100875 China

## Abstract

Understanding glacier mass balance (MB) change under global warming is important to assess the impact of glacier change on water resources. This study evaluated the applicability of a modified distributed surface energy balance model (DSEBM) with 3–h temporal and 100-m spatial resolution to the alpine Dongkemadi Glacier (DKMD) in the central Tibetan Plateau region, analyzed the causes of glacier MB variations with respect to energy balance, and evaluated MB changes under various climate scenarios. Results showed that: (i) the modified model can describe surface energy and MB of XDKMD well; (ii) net shortwave and longwave radiation, accounting for more than 80% of total heat flux, dominated the glacier energy balance during both summer and winter months; (iii) summer MB spatial patterns dominated annual MB, consistent with the fact that DKMD is a summer accumulation type glacier; and (iv) effect of increase in air temperature on glacier MB is higher than that of decrease in air temperature. The sensitivity of MB revealed by the modified DSEBM can help to understand MB changes influenced by the climate changes and to regulate water management strategies to adapt to climate changes at the catchment scale.

## Introduction

Glaciers are sensitive climate indicators^1^, and have been shrinking globally for the past decades with some localized exceptions (*e.g*., eastern Pamir Plateau and central Karakoram)^1–5^. Due to that glaciers store important water resources in the form of snow and ice (~75% of the world’s freshwater), contributing significantly to runoff, especially in mountainous areas, changes of glaciers exert a considerable influence on mountainous watershed hydrology, and indirectly have a significant and lasting impact on local and downstream ecosystems and populations^6–10^. Because of environmental lapse rates and orographic lifting (and associated cloudiness)^11^, many high-elevation catchments are energy-limited where much of the globe’s important fresh water resources are conserved^12,13^. The impacts of climate warming could vary considerably between different glaciers^14–17^, inducing different hydrological responses in glacierized mountainous basins.

The Tibetan Plateau and its surrounding area contain the largest number of the glaciers (with an area of ~100, 000 km^2^) outside the Polar Regions^4^, and 78% of them are continental^18^, which has been regarded as the Asian Water Tower and supporting 1.4 billion people^10^. Evidence showed that most of the glaciers (excluding the Karakorum) are retreating influenced by the climate changes on the Tibetan Plateau^4^. Glacier changes on the Tibetan Plateau could have affected the water discharge of large rivers^4,19,20^, glacial lake level and area^21–23^, and glacial lake outburst floods and debris flows^24–26^. In this context, the characteristics and changes in energy and mass balance of glacier on the Tibetan Plateau have drawn great attention to describe the melt processes which is used to explain the changes in glaciers^27^. An integrated assessment of glacier status (area, length and elevation) and *in situ* measurement have been conducted to understand the glacier status and mass balance on and around the Tibetan Plateau. So far 15 glaciers have undergone continuous mass balance observation^4^.

**Figure 1:**
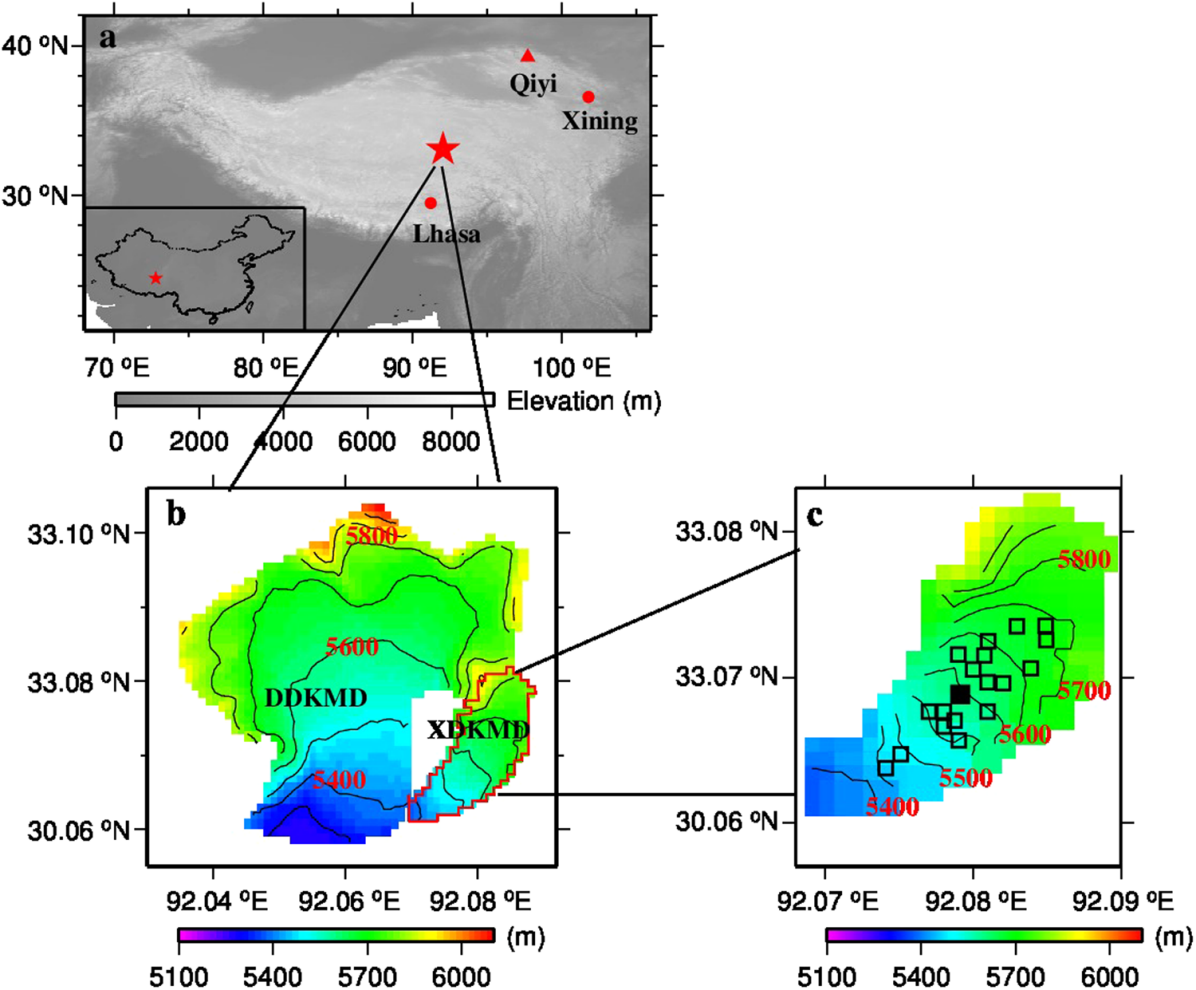
(**a**) Location of the DKMD Glacier (red star), main cities (red dots) and Qiyi Glacier referred to in discussion (red triangles); (**b**) the DKMD Glacier and its elevations; and (**c**) locations of stakes (squares) and AWS (solid square) on the XDKMD Glacier. In (**b**) and (**c**), black lines represent isoelevation contours and the red labels are the elevation above sea level. This figure was plotted using the Generic Mapping Tools (GMT) V4.5.0 https://www.soest.hawaii.edu/gmt/).

Based on the *in situ* observations of meteorology and MB on glacier surface and improvements in the understanding of physical processes of ablation and accumulation, process-based studies at point scale are crucial for process understanding and can shed light on the physics of the interaction between glaciers and climate^28,29^. Promoted by increased availability of digital terrain models and computational power, the distributed surface energy balance model (DSEBM) that takes the spatial heterogeneity of the melt process into account was developed^30^. Physical process based distributed modelling can reveal the most important variables and water balance components, as well as the locations that should be monitored^2^. Up to now, few studies provided comprehensive information of glacier mass and energy balance and its sensitivity to climate change, especially on the Tibetan Plateau (Table S1). Consequently, our objectives are: (i) to evaluate the applicability of the modified DSEBM model improved in the albedo and ground heat flux calculations; (ii) to understand and determine the drivers of glacier MB change; and (iii) to evaluate glacier MB under various climate scenarios and its sensitivity in DKMD Glacier in the central Tibetan Plateau (Fig. 1). The above three objectives will further improve the understanding of the mechanisms of change, provide a more comprehensive and systematic knowledge of the DKMD Glacier, and lay a foundation for investigating future changes in the ablation and hydrology of DKMD Glacier under a changing climate. This study will also contribute to the understanding of the overall glacier change on the Tibetan Plateau.

## Results

### Model calibration and validation in XDKMD

The calibrated parameters and their values used in DSEBM are provided in Table S2. The albedo parameters (*a*_1_ − *a*_4_, b_0_ and b_1_) were calibrated using local observations and hence differ from those for Qiyi Glacier where the formulas were developed. The air temperature lapse rate (−0.65 °C/100 m) was first calculated using gridded data for this area^31^ and then locally calibrated. The precipitation gradient with elevation was 0.01 mm/(3–h 100 m), which first adopted the value for Nyainqentanglha region (a sub-region of Tibetan Plateau including DKMD Glacier)^32^ and then was locally calibrated.

**Figure 2:**
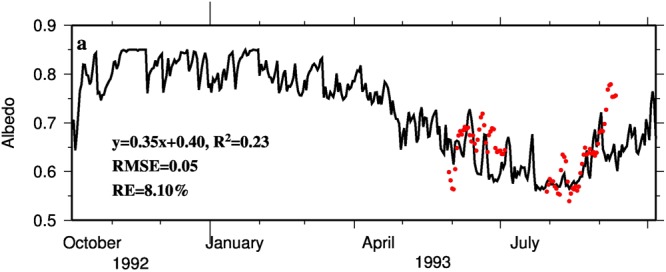
Variation of daily albedo. Red dots are *in situ* field observations made at AWS located at 5600 m shown on Fig. 1(c) and black line is simulations. The regression equation between observed and simulated albedo, *R*^2^, RMSE and relative error (RE) were presented. RE calculated by (RMSE/mean) *100%. The mean is of observed albedo.

**Figure 3:**
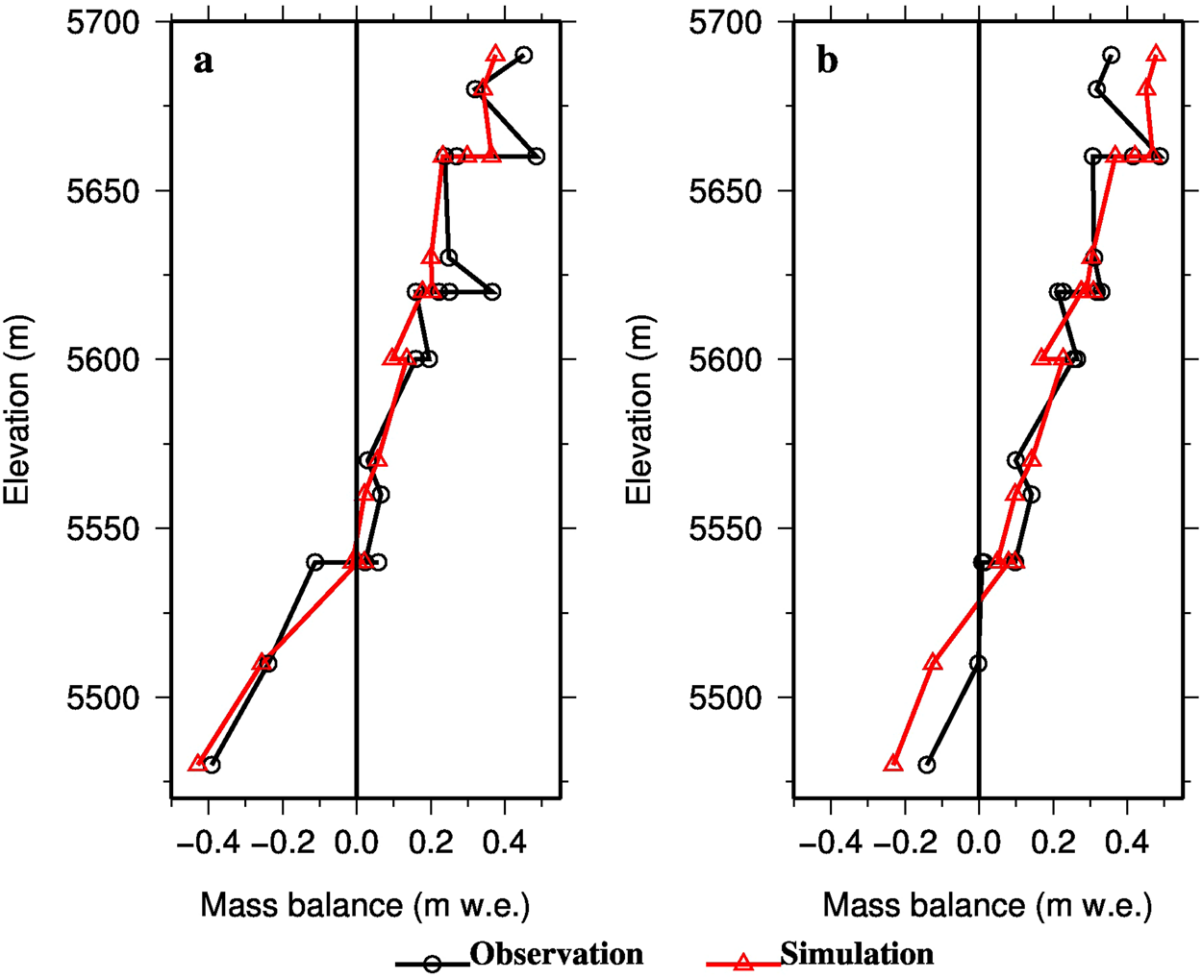
Comparison between simulated and observed glacier MB at 19 stakes on the XDKMD Glacier: (**a**) calibration period 1993; (**b**) validation period 1992. The location of the stakes is shown in Fig. 1(c).

The albedo simulation was generally acceptable for the 1993 calibration period, although it was a little low for September (Fig. 2; RMSE = 0.05 mm w.e., *R*^2^ = 0.23). The relative error was only 8.10%. Albedo decreased when air temperature increased as shown in Eqs (9–10) and Table S2. The underestimated albedo in September was caused by a rise in air temperature, which was from −6.9 °C on September 3 to −2.5 °C on September 12. The underestimation of albedo demonstrates the importance of the quality of meteorological forcing data. MB simulations at all stakes situated from 5480 m to 5690 m AMSL were acceptable for the 1993 calibration period (Fig. 3a; NS = 0.90, *R*^2^ = 0.93 and RMSE = 67.19 mm w.e.). During the 1992 validation year, the simulated MB was slightly higher than observed values at the top two stakes (at 5680 m and 5690 m AMSL) and lower at the bottom two stakes (at 5480 m and 5510 m AMSL) (Fig. 3b). Generally, the validation period simulation was reasonably good (NS = 0.80, *R*^2^ = 0.93 and RMSE = 71.14 mm w.e.).

### Mass and surface energy balance in the entire DKMD

**Figure 4:**
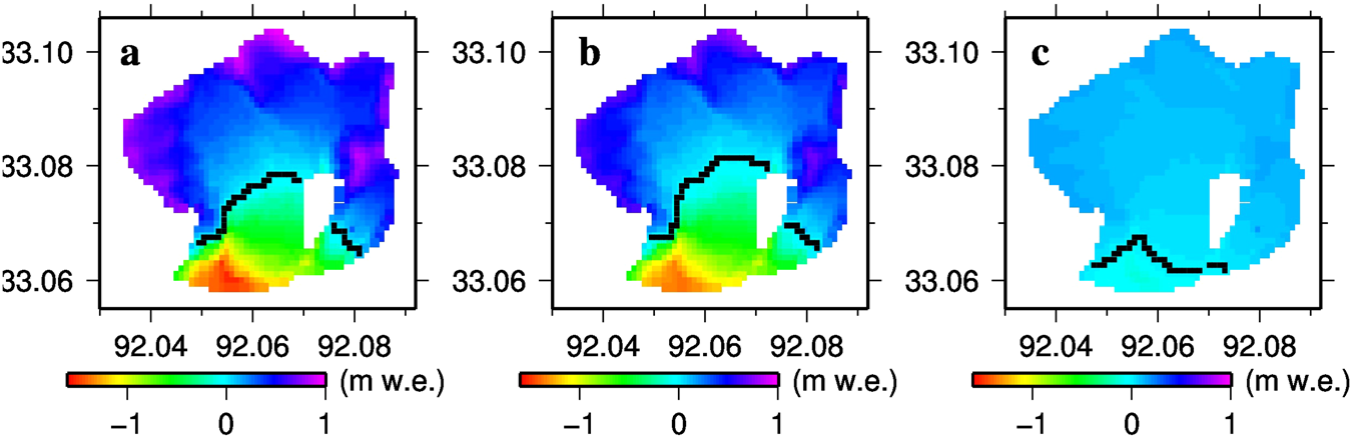
Spatial distributions in 1993 (**a**) annual, (**b**) summer, and (**c**) winter glacier MB of the DKMD Glacier. Black cells denote equilibrium lines. This figure was plotted using the Generic Mapping Tools (GMT) V4.5.0 https://www.soest.hawaii.edu/gmt/).

Taking 1993 MB for an example (Fig. 4), most of the DKMD Glacier experienced accumulation. MB for the entire glacier was 157, 68 and 88 mm w.e. for the whole year, summer and winter, respectively. Correspondingly, ELA was 5538, 5560 and 5391 m, respectively. In winter, almost the entire glacier experienced accumulation and MB varied little spatially (Fig. 4c), while in summer (Fig. 4b) and over the whole year (Fig. 4a), MB varied substantially, from about −1.4 m w.e. at the glacier tongue to greater than 0.8 m w.e. at high elevations. The spatial pattern of annual MB was similar with summer.

**Figure 5:**
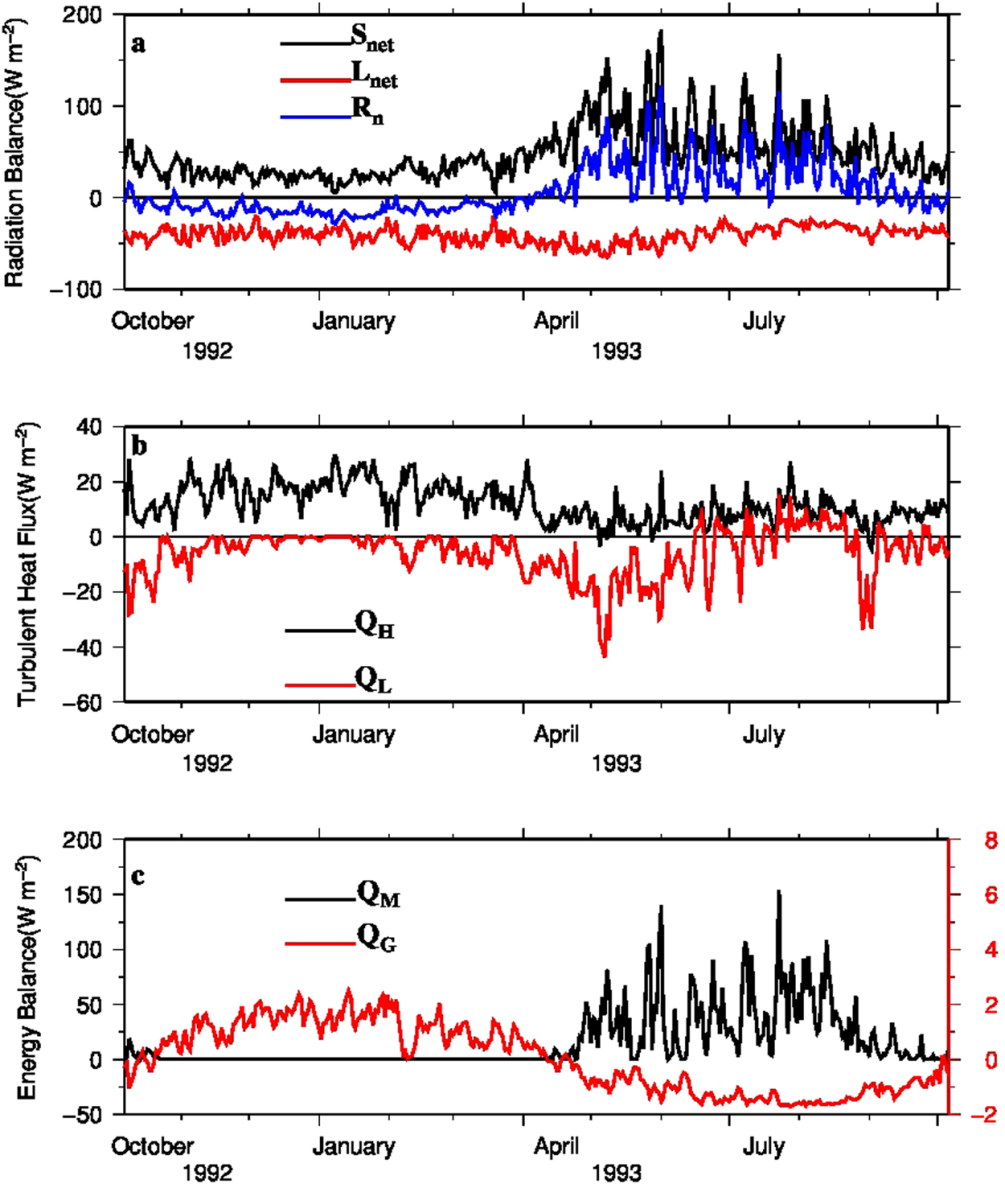
Daily energy components of DKMD Glacier in 1993. (**a**) Net shortwave radiation *S*_net_, net longwave radiation *L*_net_ and net radiation *R*_n_; (**b**) Turbulent sensible heat flux *Q*_H_ and turbulent latent heat flux *Q*_L_; (**c**) Ground heat flux *Q*_G_ and the melting component *Q*_M_.

Variabilities of daily energy components are shown in Fig. 5. Net shortwave radiation (*S*_net_) was directed towards the surface and varied largely during the year, with high values in summer (65 W m^−2^ in average) and low values in winter (34 W m^−2^ in average) (Table 1). Besides solar altitude, glacier surface albedo also played a main role in seasonal variation of *S*_net_. For the entire DKMD Glacier, albedo was 0.75 on average in winter and 0.54 on average in summer. Net longwave radiation (*L*_net_) varied less than *S*_net_ during a year (Fig. 5a), with an average of 39 W m^−2^ in summer and 42 W m^−2^ in winter, and was directed away from glacier surface. The reason is that incoming and outgoing longwave radiations have similar seasonal patterns and outgoing longwave radiation is much higher. Turbulent *Q*_H_ directed towards the glacier surface indicates that heat was transferred from air to glacier surface. *Q*_H_ was higher and more varied in winter than in summer, because of the larger difference between air temperature and surface temperature and the higher wind speed in winter than in summer (Fig. 5b). Turbulent *Q*_L_ directed away from glacier surface for most of the year, but a direction shift occurred in summer (Fig. 5b). This means vapor condensation occurred on the glacier surface, because air temperature and relative humidity in summer were high and led to a reversal of vapor pressure gradient. Net radiation (*R*_n_, *i.e*., *S*_net_ + *L*_net_) and *Q*_G_ showed different directions in winter and summer (Fig. 5b). *R*_n_ directed away from the surface in winter and towards the surface in summer (Fig. 5a), while *Q*_G_ was the opposite (Fig. 5c). *Q*_G_ was very low in both winter and summer. *Q*_R_ only occurred in summer, with values close to zero (therefore not shown in Fig. 5). *Q*_M_ was positive in summer, meaning that glacier melting occurred.

**Table 1:** Energy components and the percentage of each energy component in relation to the sum of all energy components in 1993.

	Winter (Oct. 7–May 4)		Summer (May 5–Oct. 6)		Year (Oct. 7–Oct. 6)	
	W m^−2^	%	W m^−2^	%	W m^−2^	%
*S*_net_	34↓	35↓	65↓	55↓	48↓	45↓
*L*_net_	42↑	43↑	39↑	32↑	41↑	38↑
*Q*_H_	15↓	15↓	8↓	7↓	12↓	11↓
*Q*_L_	6↑	6↑	6↑	5↑	6↑	5↑
*Q*_G_	0.9↓	1↓	1.2↑	1↑	0.1↓	0
*Q*_R_	0.0	0	0.2↓	0	0.0	0
Sum	98	100	103	100	106	100

↑ is energy flux directed away from the surface; ↓ is energy flux directed towards the surface.

As shown in Table 1 the radiation heat flux (*S*_net_ and *L*_net_) was the most important component of the energy balance and accounted for 83% of the annual heat flux together. The ratio of *S*_net_ to total energy was higher in summer while that of *L*_net_ was higher in winter. Therefore, *R*_n_ contributed towards causing glacial melt in the summer but reduced melting in the winter. Turbulent *Q*_H_ and *Q*_L_ accounted for 11% and 5% of the annual heat flux, respectively. *Q*_G_ contributed a little to the seasonal variation of energy. The contribution of *Q*_G_ and *Q*_R_ can both be neglected for annual heat flux.

### Sensitivity of mass balance in the entire DKMD

The response of MB to various scenarios of climate change showed (Table 2): (i) to some extent, increasing precipitation offset effects of increasing air temperature, and vice versa; (ii) for a certain magnitude, wetting and drying effects are roughly equivalent. E.g., when temperature remains unchanged, 20% decrease (or increase) in precipitation will cause 0.23 m w.e. decrease (or 0.22 m w.e increase) in MB; and (iii) for a certain magnitude, warming effect is higher than cooling effect. E.g., when precipitation remains unchanged, 1 °C increase in air temperature will cause 0.33 m w.e. decrease in MB, which is much higher than effect of 1 °C decrease (0.23 m w.e.). Therefore, effect of 1 °C decrease can be offset by a 20% decrease in precipitation, while to offset 1 °C warming, about a 30% increase in precipitation is required. The important reason is that the ratio of snow to precipitation will decrease/increase, when air temperature increase/decrease.

**Table 2:** Simulated changes of surface MB under different scenarios.

Scenarios	Temperature (°C)	Precipitation (%)	Change of MB (m w.e.)
1	−1	−20	0.00
2	−1	0	0.23
3	−1	20	0.45
4	0	−20	−0.23
5	0	20	0.22
6	1	−20	−0.56
7	1	0	−0.32
8	1	20	−0.11

## Discussion

### Glacier mass and surface energy balance

Summer and annual MB spatial patterns were similar, indicating the summer MB change dominance in annual MB change. This was because most precipitation (85% of annual total amount) and melting occurred in summer (see section 2.1). The similar MB for summer and winter was due to strong melting consuming most of the precipitation in summer. The similar spatial patterns of MB in summer and the whole year proved that DKMD is a summer accumulation glacier, and is much more sensitive to air temperature change in contrast with winter accumulation glaciers^33^. This is because in summer air temperature is near or above 0 °C whereas in winter air temperature is much lower than 0 °C (see section 2.1). The slight increase in air temperature in summer will facilitate the glacier melt greatly compared to the effects of equivalent absolute increase of air temperature in winter.

Seasonal variations in the melt rate of DKMD Glacier were controlled by the seasonality of the energy balance (Fig. 5c). Glacier melting occurred in summer, and energy for melting *Q*_M_ was mainly provided by *S*_net_ (Fig. 5a,c). Turbulent heat flux and *Q*_R_ also provided energy for melting, but their contributions were very little. Over all, *Q*_L_ consumed energy during the summer period, although condensation released limited energy. In winter, *L*_net_ dominated the radiation balance and led to negative *R*_n_. Although the positive turbulent heat flux, *i.e. Q*_H_, and *Q*_G_, compensated negative heat flux to some extent, not enough energy was available for melting.

### Sensitivity of MB to climate changes

As shown in Table 2, MB change reflected the complex influence of climate changes in DKMD. For DKMD Glacier, MB changed −0.21 m w.e. during melting season when air temperature increases 1 °C (in the region near ELA). Consistent with our result, a similar result were also reported by Zhang *et al*. with a MB change of −0.18 m w.e.^34^. Precipitation and air temperature are two key factors affecting glacier by controlling accumulative and melting processes, respectively^34,35^. For precipitation, change of MB from precipitation −20% to actual conditions is roughly equivalent to that from actual conditions to precipitation +20%, due to their similar effects on glacier surface (*e.g*., snow conditions and albedo), in addition to direct effect of precipitation change. Interestingly, the sensitivity of MB to air temperature varies with increasing air temperature (shown in Table 2), that is to say, absolute MB change increased with the increase in temperature when precipitation change kept constant (*e.g*., when precipitation remains changed, the absolute change of MB is 0.33 m w.e. from actual conditions to temperature +1 °C, which is higher than that from temperature −1 °C to actual conditions (0.23 m w.e.). The reason is that the altered glacier surface due to melting caused by warming has lower albedo and then obtains more energy for melting. Furthermore, historical observation from a nearby meteorological station (Tuotuohe) reveals that air temperature increased 1.37 °C and precipitation increased 13% in the past 50 years. This means that MB will most likely decrease but with high annual variability in the future, since increasing precipitation can not totally offset effect of increasing air temperature in the DKMD glacier.

### Effects of warming on MB of DKMD Glacier in contrast with Qiyi Glacier

Due to different ambient atmosphere conditions, sensitivity of glacier MB accordingly exhibited different patterns^34,35^. DKMD Glacier (located in inner Tibetan Plateau) exhibited lower sensitivity to climate change than other glaciers when comparing entire glaciers, and was relative stationary^35^. E.g., 1 °C warming will cause MB to decrease less than 0.25 m w.e. for DKMD Glacier, while will cause a MB decrease of more than 1.00 m w.e. for Qiyi Glacier (See Fig. 1a for location, a continental glacier located in middle Qilian Mountain on northeastern TP) during the two periods July 1 to October 9 and June 30 to September 5^36,37^. While, MB of DKMD Glacier is more sensitive than Qiyi Glacier to 1 °C warming in summer^34^ in comparison made in the regions near ELA of each glacier, which divides the accumulation and ablation areas and is generally considered as the most sensitive one to climate change among the glacier parameters^34,38^. The reason for the contradiction between the two comparisons lies in the compared regions (partial glacier or entire glacier), that is to say, the ratio of accumulation area to total glacier area plays a vital role. The accumulation area covers about half of DKMD Glacier, which is much larger than Qiyi Glacier with accumulation area ratio of about 15%. From this perspective, stability of DKMD Glacier induced by high ratio of accumulation area alleviates the response of glacier MB to climate warming.

### Study area, methods and data

#### Study area

As one of the only two glaciers with relatively long-term MB observational studies on Tibetan Plateau(See Supplementary Information), the DKMD Glacier, situated in the mid-Tanggula Mountains, central Tibetan Plateau region, is an alpine glacier that comprises part of the headwaters of the Yangtze River (Fig. 1a). The entire DKMD Glacier has an area of 15.87 km^2^ in 2010, extending from 5278 m to 6087 m AMSL^39,40^. The DKMD Glacier is composed of the south facing Da Dongkemadi Glacier (Da DKMD, 14.14 km^2^, and 5278–6087 m AMSL) and the southwest facing XDKMD Glacier (1.73km^2^, and 5372–5912 m AMSL) (Fig. 1b). Both Da DKMD and XDKMD have a similar elevation range, topography and climatology which justify the evaluations conducted on the XDKMD and the application of the model to the entire DKMD. The headwater region of the Yangtze River is under the influence of the Westerlies between October and April which results in an average air temperature of −11.6 °C, 20% of the annual total precipitation, and an average wind speed of 4.3 m s^−1^. The region is subjected to monsoon influences between May and September with an average air temperature of about −4 °C, 80% of the annual total precipitation, and average wind speed of 3.4 m s^−1 ^^31^.

Based on 1992–1993 Aanderaa automatic weather station (AWS) observations at 5600 m on XDKMD which is also the equilibrium line altitude (ELA) (Fig. 1c), the annual mean daily air temperature is approximately −10 °C with an annual range of −26.5 to 2.7 °C, changing dramatically with seasons. Only 38 d a^−1^ had daily mean air temperatures exceeding 0 °C, mostly occurring in August. Annual precipitation at 5500 m AMSL is approximately 909 mm, 85% of which occurred between June–September.

## Methods

The DSEBM model is a fully distributed surface energy balance model. Combined with snowfall, this model can indirectly generate mass balance by converting its energy available for melting into melt water equivalent. It computes each energy component and its contribution to glacier ablation as follows:

1 $$S\downarrow (1-\alpha )+L\downarrow +L\uparrow +{Q}_{{\rm{H}}}+{Q}_{{\rm{L}}}+{Q}_{{\rm{G}}}+{Q}_{{\rm{R}}}+{Q}_{{\rm{M}}}=0$$

where *S*↓ is incoming solar radiation; α is albedo; *L*↓ is incoming longwave radiation; *L*↑ is outgoing longwave radiation; *Q*_H_ is sensible heat flux; *Q*_E_ is latent heat flux; *Q*_G_ is ground heat flux in ice or snow; *Q*_R_ is energy supplied by rain; and *Q*_M_ is energy available for melt. The effects of subsurface melting are not considered. Energy fluxes directed towards the glacier surface are positive. Units are W m^−2^. *Q*_M_ is converted into melt water equivalent and corrected for the mass transfer by sublimation or condensation, henceforce referred to as ablation. Then combined with snowfall converted to water equivalent, mass balance is obtained.

The computations of *L*↑, *Q*_H_, *Q*_L_ and *Q*_R_ in Eq. (1) follow Hock & Holmgren^30^. Ground heat flux was calculated using a temperature profile (∂*T*/∂*z*) during a given time span, instead of linear interpolation during the entire melting period^28^. Albedo was computed using a more feasible method developed on Tibetan Plateau by Jiang *et al*.^41^. The freezing process was calculated using a simplified method. The detailed computation of the above energy component and parameter are in Supplementary Information. The air temperature used to divide snowfall and rainfall is adopted from Cuo *et al*.^31^. Precipitation is pure rainfall when air temperature > = 3.4 °C, and pure snowfall when air temperature < = 1.6 °C. Within the range1.6–3.4 °C, the proportions of snowfall and rainfall are obtained from linear interpolation.

On account of the availability of detailed observations of albedo and MB for the XDKMD Glacier, model applicability is tested on the XDKMD Glacier (Fig. 1). After the test, the model is applied to the entire DKMD Glacier. To assess the response of MB to various scenarios of climate change, eight scenarios were created with air temperature change (±1 °C) and precipitation change (±20%).

### Data

Data included observed meteorological forcing, glacier surface MB, albedo, and elevation records. Meteorological forcing data included air temperature, wind speed, relative humidity, precipitation, incoming shortwave radiation, and incoming longwave radiation. MB and albedo were used to calibrate and evaluate the model. The model was run at 3–h time interval but evaluated at a daily time step. Glacier surface mass balance year, starting from October 7 of previous calendar year and ending on October 6 of the following calendar year, was used for calculating annual statistics. Winter is from October 7 to May 4 of the following year and summer is from May 5 to October 6. Statistics for the entire glacier was obtained by averaging all the pixels representing glacier.

For meteorological forcing data, precipitation was corrected and missing data in precipitation and relative humidity were filled using linear regression interpolation, and then all observed meteorological variables from the DKMD Glacier released at daily intervals were temporally downscaled to generate 3–h forcing data (See Supplementary Information).

Glacier albedo (from Fujita & Ageta^42^) was monitored from May 30 to September 11, 1993 at 5600 m AMSL on XDKMD. Glacier surface MB, originally from Fujita & Ageta^42^, was observed for 1992–1993 using 27 stakes when the AWS was running, distributed in both accumulation and ablation zones on XDKMD Glacier (Fig. 1c). Among these stakes, 19 stakes in the accumulation and ablation zones covering an elevation range of 5480–5690 m, had complete records and were selected to calibrate and validate the model. Albedo and glacier MB measured by stakes in 1993 were used to calibrate the model and glacier MB measured by stakes in 1992 was used to validate the model.

The 90 m digital elevation model (DEM) from the Shuttle Radar Topography Mission (SRTM) was interpolated using cubic convolution to generate a 100 m DEM, the spatial resolution of the model. Glacier area from GLIMS glacier database (around 1970)^39^ was used as an initial glacier condition for the simulation period 1992–1993 which was justified by the slow glacier change before 1990s and dramatic change after 1990s^40^. The 0.003768° (400 m) glacier map from the GLIMS database was also converted to a 100 m map to match the model DEM resolution and to obtain the spatial distribution of the glacier in the model. The glacier map was based on materials in 1970 and therefore corrected according to the field trip in 1993.

## Electronic supplementary material

Supplementary Information
