# Supplementary material for: The energy and mass balance of a continental glacier: Dongkemadi Glacier in central Tibetan Plateau
Source: Sci Rep. 2018 Aug 24;8:12788. doi: 10.1038/s41598-018-31228-5 (PMC6109143; doi:10.1038/s41598-018-31228-5)
Supplement: Supplementary file 1 — Supplementary Information [file 41598_2018_31228_MOESM1_ESM.docx]

### The energy and mass balance of a continental glacier: Dongkemadi Glacier in central Tibetan Plateau

Liqiao Liang, Lan Cuo & Qiang Liu

### Author information

### Affiliations

1. Key Laboratory of Tibetan Environment Changes and Land Surface Processes, Institute of Tibetan Plateau Research, Chinese Academy of Sciences, Beijing, China

Liqiao Liang, Lan Cuo

2. Center for Excellence in Tibetan Plateau Earth Sciences, Beijing, China

Liqiao Liang, Lan Cuo

3. State Key Laboratory of Water Environment Simulation, School of Environment, Beijing Normal University, Beijing 100875, China

Qiang Liu

### Corresponding author

Correspondence to Lan Cuo

Why choose DKMD Glacier as a study area?

Glacier observations on the Tibetan Plateau have been limited by the harsh environment and difficult access. So far only fifteen out of ~36,800 glaciers on the Tibetan Plateau are currently undergoing glaciers mass balance (MB) observations, and most of these observations were initiated around 2000^4^. Only the Qiyi and Xiao Dongkemadi (XDKMD, a branch of the entire Dongkemadi) glaciers have undergone relatively long-term MB observational studies, starting in 1975 (19-year record with 21 years missing) and 1989 (26-year record), respectively. Of the two glaciers, only the XDKMD Glacier has a continuous record of observation up to the present day. Meteorological observations on glacier surfaces are also scarce on the Tibetan Plateau. During 1992–1993, the XDKMD Glacier was briefly observed for air temperature, precipitation, wind speed, relative humidity, and incoming shortwave radiation^42^, providing an opportunity to study glacier energy and mass balance. To date, much research has been conducted on the DKMD Glacier, such as monitoring glacier thickness^43, 44^, analyzing spatial variation in albedo^45^, detecting snow line locations^46^, analyzing changes in snow coverage^47^, conducting runoff simulations by means of conceptual hydrological modeling^40^, snowmelt^48^, and glacier surface MB modeling focused on the XDKMD Glacier^42, 49, 50, 51^. However, changes of mass and energy balance for the entire DKMD Glacier are still not well understood.

Computation of energy components and main parameters

According to Zhang *et al*.^28^, ground heat flux is calculated using a temperature profile (∂*T*/∂*z*) during a given time span, instead of linear interpolation during the entire melting period. Temperature of ice is close to a constant at the bottom of active layer^52^, using the annual average surface temperature in this study.

$Q_{\text{G}}=K\frac{\partial T}{\partial z}$ (S1)

$K_{\text{s}}=0.029\left(1+10^{-4}\rho_{\text{s}}^{2}\right)$ (S2)

$K_{\text{i}}=\frac{488.2}{273.2+T_{\text{z}}}+0.47$ (S3)

where *K* is thermal conductivity (W m^–1^ ^o^C^–1^), and can be *K_s_* or *K_i_*, depending on the surface condition. *K*_s_ is for snow^53^ and *K*_i_ is for ice^54^; *ρ*_s_ is snow density (kg m^–3^); *T*_z_ is temperature at depth *z* (m).

Surface temperature is assumed to be zero if energy is available for melt (*i.e*., *Q*_M_ >0). If there is no melt, energy becomes negative, surface temperature is lowered iteratively by an interval of 0.2 K (0.25 K in Hock & Holmgreen^30^) until energy is balanced. According to Fujita *et al*.^49^, 20% melting water is assumed to be preserved at the snow-ice boundary due to refreezing. And this amount of water is excluded from the calculated melting water. The calculation of released energy by refreezing is simplified in the iteration process.

Jiang *et al.*^40^ demonstrated that the DSEBM model generally calculated snow albedo unsuccessfully on the Qiyi Glacier on the Tibetan Plateau region. They subsequently developed new snow albedo formulas by incorporating time after snowfall events, air temperature, and level of cloudiness. Compared to the original model, here snow albedo is independent of the former time step. Ice albedo was considered solely a function of air temperature instead of being a constant. Simulation by these formulas was in good agreement with observations on the Qiyi Glacier in 2007 with a correlation coefficient between simulated and observed glacier albedo of 0.97 and a root-mean-square error of 0.05. Hence, the albedo formulas developed by Jiang *et al.*^40^ were used here. Snow and ice albedo were calculated as follows:

Snow: $\alpha_{\text{s}}=a_{1}T+a_{2}\exp(a_{3}\sqrt{n_{\text{d}}})+a_{4}\frac{S\downarrow}{I_{\text{TOA}}}$ (S4)

Ice: $\alpha_{\text{i}}=b_{0}+b_{1}T$ (S5)

where *T* is air temperature (^o^C); *n*_d_ is the number of days after snowfall; *a*_1_–*a*_4_ and *b*_0_ and *b*_1_ are empirical parameters; and *S*↓/*I*_TOA_ is the ratio of incoming shortwave radiation to direct solar radiation at the top of the atmosphere, used to reflect the influence of clouds.

Sky emissivity for calculating incoming longwave radiation was derived from the product of vapor pressure, air temperature, and cloudiness^55^. Sky emissivity function is:

$\varepsilon=0.7+0.0000595\frac{e_{\text{z}}}{100}\exp\left(\frac{1500}{T_{\text{z}}}\right)$. (S6)

The air temperature used to divide snowfall and rainfall is adopted from Cuo *et al.*^31^, which is obtained using hourly air temperature, snowfall, and rainfall records at 44 stations in the Northern Tibetan Plateau. Precipitation is pure rainfall when air temperature >= 3.4 ^o^C, and pure snowfall when air temperature <= 1.6 ^o^C. Mixed snowfall and rainfall happens between 1.6 ^o^C and 3.4 ^o^C. Within this temperature range, the proportions of snowfall and rainfall are obtained from linear interpolation.

Data processing and downscaling

Daily air temperature, wind speed, relative humidity, and incoming shortwave radiation were observed between October 1991, and October 1993, using an Aanderaa AWS (33°04′ N, 92°03′ E; Fig. 1c) positioned on the XDKMD Glacier at 5600 m AMSL. Meteorological variables were monitored at 10 min intervals between May 20 and September 15 for each year and at 1–h intervals at other times. Precipitation was recorded twice daily at 5500 m AMSL using a Chinese-type rain gauge and tipping-type rain gauge (not having a wind shield), and precipitation data were corrected based on Tretyakov-type gauge observations^56^ (having a wind shield meeting WMO standard^57^). Wind-induced under catch, trace amounts of precipitation, and wetting loss were further corrected using the method of He *et al.*^58^. Relative humidity records were started from May 1992, but precipitation was only observed in the summer of 1993. Using linear regression interpolation, any missing relative humidity and precipitation data during the observation period were filled by data obtained from Tuotuohe, the nearest meteorological station (34.22° N, 92.43° E; 4533 m AMSL; 60 km from the DKMD Glacier), and a gridded climate dataset generated by Cuo *et al.*^31^ that covers the region, respectively.

All observed meteorological variables from the DKMD Glacier were released at daily intervals and were temporally downscaled to generate 3–h forcing data. Precipitation was evenly distributed throughout the day. Wind speed and relative humidity remained unchanged during the day. Daily air temperature was downscaled to 3–h air temperature using Hermite interpolation^59^, which takes into account diurnal temperature ranges and the timing of daily maximum and minimum air temperatures. The squared relative coefficient (*R*^2^) between observed daily air temperature and daily air temperature averaged from calculated 3–h air temperature during the modeling period was 0.999. Based on the relationship developed by Bristow & Campbell^60^, daily incoming shortwave radiation was downscaled to 3–h time step using 3–h varied clear-sky transmissivity adjusted by the daily temperature range. The downscaled 3–h incoming shortwave radiation was corrected by multiplying the ratio of the observed value to the calculated value during observation period. The calculated daily incoming shortwave radiation was smoother than observation with *R*^2^=0.42. Three-hour incoming longwave radiation was calculated by 3–h interval of air temperature, emissivity and cloud factors using Eq. (10) by Hock and Holmgren^30^. Sky emissivity was calculated by Eq. (S6).

Supplementary References

(for references 1 to 42 see main text)

43. Li, Z., Xing, Q., Liu, S.Y., Zhou, J.M. & Huang, L. Monitoring thickness and volume changes of the Dongkemadi Ice Field on the Qinghai-Tibetan Plateau (1969–2000) using Shuttle Radar Topography Mission and map data. *Int. J. Digit. Earth* **5(6)**, 516–532, (2012).

44. Ke, L.H., Ding X.L. & Song, C.Q. Estimation of mass balance of Dongkemadi glaciers with multiple methods based on multi-mission satellite data. *Quatern. Int*. **371**, 58–66, (2015).

45. Wang, J., He, X.B., Ye, B.S. & Yang, G.J. Variations of albedo on the Dongkamadi Glacier, Tanggula Range. *J. Glaciol. Geocryology* **34(1)**, 21–28, (2012).

46. Huang, L., *et al.* Classification and snow line detection for glacial areas using the polarimetric SAR image. *Remote Sens. Environ.* **115(7)**, 1721–1732, (2011).

47. Liu, J.F., Yang, J.P., Yang, Y. & Chen, R.S. Annual variations of snow cover and its relation to air temperature and precipitation in Dongkemadi river basin in the source regions of the Yangtze River. *J. Glaciol. Geocryology* **29(6)**, 862–868, (2007).

48. Liu, J.F., Yang, J.P., Chen, R.S. & Yang, Y. The Simulation of Snowmelt Runoff Model in the Dongkemadi River Basin, Headwater of the Yangtze River. *Acta Geographica Sinica* **61(11)**, 1149–1159, (2006).

49. Fujita, K., Ohta, T. & Ageta, Y, Characteristics and climatic sensitivities of runoff from a cold-type glacier on the Tibetan Plateau. *Hydrol. Process.* **21(21)**, 2882–2891, (2007).

50. Fujita, K., Ageta, Y., Pu, J.C., & Yao, T.D. Mass balance of Xiao Dongkemadi glacier on the central Tibetan Plateau from 1989 to 1995. *Ann. Glaciol*. **31(1)**, 159–163, (2000).

51. Pu, J.C., *et al*. Rapid decrease of mass balance observed in the Xiao (Lesser) Dongkemadi Glacier, in the central Tibetan Plateau. *Hydrol. Process.* **22(16)**, 2953–2958, (2008).

52. Huang, M.H., Wang, Z.X. & Ren, J.W. Ice temperature of glaciers in China. *J. Glaciol. Geocryology* **4(1)**, 20–28, (1982).

53. Mellor, M. Engineering properties of snow. *J. Glaciol.* **19(81)**, 15–66. (1977).

54. Hobbs, P.V. *Ice physics*. Clarendon Press, Oxford. (1974).

55. Idso, S.B. A set of equations for full spectrum and 8- to 14 µm and 10.5- to 12.5 µm thermal radiation from cloudless skies. *Water Resour. Res*. **17(2)**: 295–304, (1981).

56. Ueno, K., *et al*. Characteristics of precipitation distribution in Tanggula, Monsoon, 1993. *Bull. Glacier Res.* **12**, 39–47, (1994).

57. Ueno, K., Shiraiwa, T. & Yamada, T. Precipitation environmentin the Langtang Valley, Nepal Himalayas. *Snow and Glacier Hydro1ogy, IAHSPubl*. **218**, 207–219, (1993).

58. He, X.B., Ye, B.S. & Ding, Y.J. Bias correction for precipitation measurement in Tanggula Mountain Tibetan Plateau. *Adv. Water Sci.* **20(3)**, 403–408, (2009).

59. Conte, S.C. & de Boor, C. *Elementary Numerical Analysis, and algorithmic apporoach. International Series in Pure and Applied Mathematics, 3^rd^ Edition*, McGraw-Hill Book Company, New York. (1980).

60. Bristow, K.L. & Campbell, G.S. On the relationship between incoming solar radiation and daily maximum and minimum temperature. *Agric. For. Meteorol.* **31(2)**, 159–166, (1984).

61. Klok, E.J. & Oerlemans. J. Model study of the spatial distribution of the energy and mass balance of Morteratschgletscher, Switzerland. *J. Glaciol.* **48(163)**, 505–518, (2002).

62. Gerbaux, M., Dedieu, J.P., Etchevers, P., Vincent, C. & Genthon, P. Surface mass balance of glaciers in the French Alps: distributed modeling and sensitivity to climate change. *J. Glacial.* **51(175)**, 561–572, (2005).

63. Reid, T.D., Carenzo, M., Pellicciotti, F. & Brock, B.W. Including debris cover effects in a distributed model of glacier ablation. *J. Geophys. Res.: Atmos.* **117(D18)**, 119–130, (2012).

64. M**ö**lg, T., Maussion, F., Yang, W. & Scherer, D. The footprint of Asian monsoon dynamics in the mass and energy balance of a Tibetan glacier. *Cryosphere* **6(6)**, 1445–1461, (2012).

65. Lu, C.X., Wang, L., Xie, G.D. & Leng, Y.F. Altitude effect of precipitation and spatial distribution of Qinghai-Tibetan Plateau. *J. Mt. Sci.* **25(6)**, 655–653, (2007).

Supplementary Tables

Supplementary Table S1 Summary of relevant glacier energy and mass balance modelling (the current paper is added for completeness). Not all papers performed the following key results and these are denoted with a N/A representing ‘not applicable’ in the relevant part of the ‘Key results’ column. In the ‘Key results’ column three components are identified by the code: (1) MB or melt rates and calibration; (2) causes of glacier MB change due to specific energy flux; and (3) sensitivity of glacier MB to climate change.

| Study | Model | Location/type/study size | Key results |
| --- | --- | --- | --- |
| Klok & Oerlemans^61^ | Distributed | Morteratschgletscher in the southeast Switzerland/Maritime/17.5 km^2^ | (1) Model was calibrated by observed MB, albedo, snow depth and melt at stakes. |
|  |  |  | (2) Proportion of L_net_ increased and that of S_net_ decreased with increasing elevation. |
|  |  |  | (3) 1 ˚C warming results in a change in MB of -0.7 m w.e. |
| Hock & Holmgren^30^ | Distributed | Storglaciären in northern Sweden/Maritime/3 km^2^ | (1) High ablation was underestimated and low ablation was overestimated. Model was calibrated by observed MB and discharge (R^2^=0.85). |
|  |  |  | (2) Net radiation only contributed roughly 40% and 60% of the melt energy in 1993 and 1994. |
|  |  |  | (3) N/A |
| Gerbaux *et al.*^62^ | Distributed | de Saint-Sorlin and d’Argentière, French Alps/Maritime/3 and 13 km^2^, respectively | (1) Both glaciers lost mass in the last 23 years. Model was calibrated by satellite-derived elevation of the snowline. |
|  |  |  | (2) N/A |
|  |  |  | (3) MB was more sensitive to climate change in the ablation than in the accumulation area. |
| Jiang *et al*.^36^ | Distributed | Qiyi on the North margin of Tibetan Plateau/Continental/2.87 km^2^ | (1) The model simulated MB well in summer 2008 (R=0.783). Model was calibrated by observed snowline and MB. |
|  |  |  | (2) N/A |
|  |  |  | (3) 1 ˚C warming results in a change in MB of -1.02 m w.e. |
| Reid *et al.*^63^ | Distributed | Haut Glacier d’Arolla, Switzerland/Maritime/5.3 km^2^ | (1) Debris cover reduced glacier melt in summer 2010. Model was calibrated by observed MB. |
|  |  |  | (2) N/A |
|  |  |  | (3) N/A |
| Mölg *et al.*^64^ | Distributed | Zhadang, central Tibetan Plateau/Continental/2.0 km^2^ | (1) Vertical MB profile is more determined by elevation than by MB in a specific year. Model was calibrated by observed surface height change and surface temperature at AWS and MB at stakes. |
|  |  |  | (2) Temporal energy pattern for period April-June varied between different years. |
|  |  |  | (3) N/A |
| Fujita &  Ageta^50^ | Elevation bands | Xiao Dongkemadi, central Tibetan Plateau/Continental/1.8 km^2^ | (1) Model was calibrated by observed MB at stakes, and albedo and ice temperature. |
|  |  |  | (2) N/A |
|  |  |  | (3) MB is more sensitive to air temperature than to other variables, such as precipitation, solar radiation, relative humidity and wind speed. |
| Fujita *et al*.^49^ | Distributed | Dongkemadi, central Tibetan Plateau/Continental/15.87 km^2^ | (1) Model was calibrated by observed MB at stakes, and albedo and ice temperature. |
|  |  |  | (2) N/A |
|  |  |  | (3) N/A |
| This study | Distributed | Dongkemadi, central Tibetan Plateau/Continental/15.87 km^2^ | (1) Model was calibrated by observed MB at stakes and albedo. |
|  |  |  | (2) Analyzes the causes of glacier mass balance change. |
|  |  |  | (3) Sensitivity of glacier MB to climate change, and comparing sensitivity to warming with other glaciers worldwide. |

Table S2 Optimized parameter values for the DSEBM model in the XDKMD Glacier

| Parameter | Value | Units | Source |
| --- | --- | --- | --- |
| Air temperature lapse rate | −0.65 | ^o^C/100 m | Cuo *et al*.^31^ with local calibration |
| Precipitation gradient with elevation | 0.01 | mm/(3–h 100 m) | Lu *et al.*^32^with local calibration |
| Roughness length of wind | 0.0027 | m | Hock & Holmgren^30^ with local calibration |
| Roughness length of temperature | 0.000027 | m | local calibration |
| Roughness length of water vapor | 0.000027 | m | local calibration |
| Albedo parameter *a*_1_ | −0.013 |  | local calibration |
| Albedo parameter *a*_2_ | 0.59 |  | local calibration |
| Albedo parameter *a*_3_ | 0.03 |  | local calibration |
| Albedo parameter *a*_4_ | −0.186 |  | local calibration |
| Albedo parameter *b*_0_ | 0.324 |  | Jiang *et al*.^41^ |
| Albedo parameter *b*_1_ | −0.018 |  | Same as above |
